# Supplementary material for: A STING–CASM–GABARAP pathway activates LRRK2 at lysosomes
Source: J Cell Biol. 2025 Jan 15;224(2):e202310150. doi: 10.1083/jcb.202310150 (PMC11734622; doi:10.1083/jcb.202310150)
Supplement: Table S1 — shows the summary of cell lines used in this study. [file jcb_202310150_tables1.docx]

**Table S1: Summary of cell lines used in this study.**

| Cell Line | Genotype | Reference | RRID |
| --- | --- | --- | --- |
| RAW 264.7 | WT LRRK2 parental | ATCC SC-6003 | CVCL_UL71 |
| RAW 264.7 | LRRK2 KO | ATCC SC-6004 | CVCL_UL72 |
| RAW 264.7 | LRRK2 T1348N | ATCC SC-6005 | CVCL_UL73 |
| RAW 264.7 | STING KO | Talaia et al 2024 | CVCL_D7F3 |
| RAW 264.7 | STING KO + mouse STING (WT) | This paper | CVCL_D7F4 |
| RAW 264.7 | STING KO + mouse STING (amino acids 1-339) | This paper | CVCL_D7F5 |
| RAW 264.7 | TBK1 KO | This paper | CVCL_D7F6 |
| RAW 264.7 | IKKε KO | This paper | CVCL_D7F1 |
| RAW 264.7 | TBK1 KO + IKKε KO | Talaia et al 2024 | CVCL_D7F7 |
| RAW 264.7 | Atg16L1 KO | This paper | CVCL_D7EZ |
| RAW 264.7 | FIP200 KO | This paper | CVCL_E2WW |
| RAW 264.7 | mCherry-SopF | This paper | CVCL_D7F2 |
| RAW 264.7 | GABARAP KO | This paper | CVCL_D7F0 |
| RAW 264.7 | GABARAP KO + HA-human GABARAP | This paper | CVCL_E2WX |
| RAW 264.7 | LRRK2 KO + HALO-human LRRK2 | This paper | CVCL_D7F8 |
| RAW 264.7 | LRRK2 KO + HA-human GABARAP | This paper | CVCL_E2WY |
| RAW 264.7 | LRRK2 KO + HALO-human LRRK2 + HA-human GABARAP | This paper | CVCL_D7F9 |
| RAW 264.7 | LRRK2 KO + HALO-human LRRK2 (LIR1 mutant) + HA-human GABARAP | This paper | CVCL_E2WZ |
| RAW 264.7 | LRRK2 KO + HALO-human LRRK2 (LIR2 mutant) + HA-human GABARAP | This paper | CVCL_E2X0 |
| RAW 264.7 | LRRK2 KO + HALO-human LRRK2 (LIR1+2 mutant) + HA-human GABARAP | This paper | CVCL_E2X1 |
| IPSC A18945 |  |  | CVCL_RM92 |
