# Supplementary material for: A STING–CASM–GABARAP pathway activates LRRK2 at lysosomes
Source: J Cell Biol. 2025 Jan 15;224(2):e202310150. doi: 10.1083/jcb.202310150 (PMC11734622; doi:10.1083/jcb.202310150)
Supplement: Table S2 — shows the summary of supplies for cell culture, drug treatments, and lab chemicals. [file jcb_202310150_tables2.docx]

**Table S2: Summary of supplies for cell culture, drug treatments and lab chemicals**

| **Cell culture reagents** | | |
| --- | --- | --- |
| **Reagent** | **Company** | **Product Number** |
| DMEM | Thermo Fisher Scientific | 11965-092 |
| E8 | Thermo Fisher Scientific | A15169-01 |
| E8 Supplement | Thermo Fisher Scientific | A15171-01 |
| RPMI | Thermo Fisher Scientific | 11875093 |
| Rock inhibitor | Stemcell Technologies | 100-1044 |
| STEMdiff Hematopoietic Kit | Stemcell Technologies | 5310 |
| Matrigel | Corning | 356230 |
| Recombinant Human M-CSF | Peprotech | 300-25 |
| FBS | Thermo Fisher Scientific | 16140-071 |
| PBS | Thermo Fisher Scientific | 10010023 |
| Cell Stripper | Corning | 25056CI |
| Penicillin/Streptomicin (10,000 U/mL) | Thermo Fisher Scientific | 15140122 |
| Blasticidin | Invivogen | Ant-bl-05 |
| Puromycin | Thermo Fisher Scientific | A11138-03 |
| Opti-Mem | Thermo Fisher Scientific | 31985062 |
| Lipofectamine 2000 | Invitrogen | 11668019 |
| Lipofectamine RNAiMAX | Invitrogen | 2448190 |
| Lipofectamine CRISPRMAX | Invitrogen | CMAX00003 |
| Fugene HD | Promega | E2311 |
|  | | |
| **Drugs/Compounds** | | |
| **Compound** | **Company** | **Product Number** |
| 2,3 cGAMP | Chemietek | CT-CGMAP |
| DMXAA | Cayman Chemicals | 14617 |
| diABZI | Cayman Chemicals | 28054 |
| MLi-2 | Abcam | Ab254528 |
| GZD-824 | Cayman Chemicals | 21508 |
| SAR405 | Selleck Chemicals | S7682 |
| Nigericin | Cayman Chemicals | 11437 |
| ML-SA1 | Cayman Chemicals | 29958 |
| Saliphenylhalamide | Omm Scientific | N/A |
| Folimycin | Abcam | Ab144277 |
| N-Ethylmaleimide | Sigma-Aldrich | E3876 |
|  | | |
| **Lab Supplies** | | |
| **Reagent** | **Company** | **Product Number** |
| Potassium Phosphate Monobasic | J.T. Baker | 3246-01 |
| Sodium Phosphate Dibasic | J.T. Baker | 3828-05 |
| Glycine | American Bio | AB00730-05000 |
| Tris | American Bio | AB02000-05000 |
| NaCl | Sigma-Aldrich | 3624-05 |
| Hydrochloric Acid | J.T. Baker | 9535 |
| SDS | American Bio | AB01920-00500 |
| EDTA | Sigma-Aldrich | 03690 |
| Triton X-100 | Sigma-Aldrich | X100 |
| Tween-20 | Sigma-Aldrich | P7949 |
| Glycerol | American Bio | AB00751 |
| Bromphenol Blue | Sigma-Aldrich | B5525 |
| B-mercaptoethanol | Sigma-Aldrich | M3148 |
| Sucrose | Sigma-Aldrich | S0389 |
| EGTA | Sigma-Aldrich | E4378 |
| HEPES (pH 7.4) | Thermo Fisher Scientific | 15630-080 |
| DMSO | Sigma-Aldrich | D2650 |
| COmplete mini EDTA Free | Roche | 11836170001 |
| PhosSTOP | Roche | 4906837001 |
| Coomassie Plus Protein Assay Reagent | Thermo Fisher Scientific | 23236 |
| PAGEruler Plus Prestained Protein Ladder | Thermo Fisher Scientific | 26620 |
| Biotin Protein Ladder | Cell Signaling | 7727L |
| 4-15% MiniPROTEAN 10-well | Biorad | 4568084g |
| 4-15% MiniPROTEAN 12-well | Biorad | 4568085 |
| 4-15% MiniPROTEAN 15-well | Biorad | 4568086 |
| BSA | Sigma-Aldrich | A9647 |
| Non-Fat Dry Milk Omniblock | American Bio | AB10109-01000 |
| 0.45 um Nitrocellulose Membrane | Thermo Fisher Scientific | 1620115 |
| Whatman Filter Paper | VWR | 28298-020 |
| SuperSignal West Pico PLUS Chemiluminescence Substrate | Thermo Fisher Scientific | 34580 |
| SuperSignal West Femto Maximum Sensitivity Substrate | Thermo Fisher Scientific | 34095 |
| Methanol | Sigma-Aldrich | 179337-4L-PB |
| Ethanol | Decon Laboratories | 2716 |
| Ampicillin | Sigma-Aldrich | A0166 |
| Tryptone | RPI | T600-60 |
| LB + Ampicillin (100 µg/mL) | Recombinant Technologies | 760100 |
| Iron (II) Chloride | Sigma-Aldrich | 220299 |
| Iron (III) Chloride | Sigma-Aldrich | 157740 |
| Ammonium hydroxide (30%) | Sigma-Aldrich | 320145 |
| Dextran | Sigma-Aldrich | D1662 |
| Snakeskin dialysis tubing (10,000 Mol Wt) | Thermo Fisher Scientific | 68100, 10,000 |
| LS Columns | Miltenyi Biotec | 130-042-401 |
| QuadroMACS Separator | Miltenyi Biotec | 130-091-051 |
| Pierce Anti-HA Magnetics Beads | Thermo Fisher Scientific | 88837 |
| Saponin Quilajja sp. | Sigma-Aldrich | S4521 |
| Paraformaldehyde | Electron Microscopy Sciences | 19202 |
| Sodium dihydrogen phosphate monohydrate | J.T. Baker | 3818 |
| Sodium phosphate, dibasic, anhydrous | J.T. Baker | 3828 |
| ProLong™ Gold Antifade Mountant with DNA Stain DAPI | Thermo Fisher Scientific | P36935 |
| Fisherbrand™ Superfrost™ Disposable Microscope Slides | Thermo Fisher Scientific | 12-550-143 |
| Microscope Cover Slips (12 mm) | Carolina Biological Supply | 633029 |
|  | | |
| **Molecular Biology Reagents** | | |
| **Reagent** | **Company** | **Product Number** |
| Q5 High-Fidelity 2X Master Mix | NEB | M0492S |
| HIFI DNA Assembly Master Mix | NEB | E2621L |
| One-Shot STABL3 | Invitrogen | C7373-03 |
|  | | |
| **Software** | | |
| **Version** | **Company** | **RRID** |
| Prism 10 | Graphpad | SCR_002798 |
| 2.14.0/1.54f | FIJI | SCR_002285 |
| 1.7.1 | ChimeraX |  |
| AlphaFold 2.2.4 | Google Deepmind |  |
| AlphaFold Server | Google Deepmind |  |
| ChatGPT-4o | OpenAI |  |
