# Supplementary material for: A STING–CASM–GABARAP pathway activates LRRK2 at lysosomes
Source: J Cell Biol. 2025 Jan 15;224(2):e202310150. doi: 10.1083/jcb.202310150 (PMC11734622; doi:10.1083/jcb.202310150)
Supplement: Table S3 — shows the summary of plasmids used in this study. [file jcb_202310150_tables3.docx]

**Table S3: Summary of plasmids used in this study**

| **Plasmid** | **Reference** | **RRID** |
| --- | --- | --- |
| pPB-EF1A-HALO-hLRRK2 | This paper | In Progress |
| pPB-EF1A-mSTING | This paper | In Progress |
| pPB-EF1A-mSTING (1-339) | This paper | In Progress |
| pPB-HA-hGABARAP | This paper | In Progress |
| pPB-EF1A-HALO-hLRRK2 (LIR1 mutant WEVL -> AEVA) | This paper | In Progress |
| pPB-EF1A-HALO-hLRRK2 (LIR2 mutant WTFI -> ATFA) | This paper | In Progress |
| pPB-EF1A-HALO-hLRRK2 (LIR1+2 mutant WEVL -> AEVA / WTFI -> ATFA) | This paper | In Progress |
| mCherry-SopF | Addgene (135174) | Addgene_135174 |
| pPB-mCherry-SopF | This paper | In Progress |
| pEIF1a-Piggybac transposase | Michael Ward (NINDS)(Pantazis et al., 2022) |  |
