# Supplementary material for: A STING–CASM–GABARAP pathway activates LRRK2 at lysosomes
Source: J Cell Biol. 2025 Jan 15;224(2):e202310150. doi: 10.1083/jcb.202310150 (PMC11734622; doi:10.1083/jcb.202310150)
Supplement: Table S4 — shows the sequences of oligonucleotide primers used in this study. [file jcb_202310150_tables4.docx]

**Table S4: Sequences of oligonucleotide primers used in this study**

| **Primer** | **Primer** | **Reference** |
| --- | --- | --- |
| mSTING (1-339)_F | TGAACCCAGCTTTCTTGTAC | This paper |
| mSTING (1-339)_R | CTCCTCCTTTTCTTCCTG | This paper |
| MCherry-SopF Ins_F | tccatttcaggtgtcgtgacGGTTTAGTGAACCGTCAG | This paper |
| MCherry-SopF Ins_R | tttgtacaagaaagctgggtTCAATATAATATTATGCAGTCTCTATTAAG | This paper |
