# Supplementary material for: A STING–CASM–GABARAP pathway activates LRRK2 at lysosomes
Source: J Cell Biol. 2025 Jan 15;224(2):e202310150. doi: 10.1083/jcb.202310150 (PMC11734622; doi:10.1083/jcb.202310150)
Supplement: Table S5 — shows the description of antibodies used in this study. [file jcb_202310150_tables5.docx]

**Table S5: Description of antibodies used in this study**

| **Antibodies** | | | | |
| --- | --- | --- | --- | --- |
| **Antibody** | **Company** | **Product Number** | C**oncentration** | **RRID** |
| LRRK2 | Abcam | Ab133474 | 1:2000 | AB_2713963 |
| STING | Cell Signaling Technologies | 13647S | 1:1000 | AB_2732796 |
| P-STING S365 | Cell Signaling Technologies | 72971S | 1:1000 | AB_2799831 |
| P-STING S366 | Cell Signaling Technologies | 19781S | 1:1000 | AB_2737062 |
| TBK1 | Cell Signaling Technologies | 3504S | 1:2000 | AB_2255663 |
| P-TBK1 S172 | Cell Signaling Technologies | 5483S | 1:2000 | AB_10693472 |
| IKKε | Cell Signaling Technologies | 2905S | 1:2000 | AB_1147662 |
| Rab10 | Cell Signaling Technologies | 8127S | 1:1000 | AB_10828219 |
| P-Rab10 T73 | Abcam | Ab241060 | 1:1000 | AB_2884876 |
| Rab12 | Santa Cruz | Sc-515613 | 1:500 | AB_3101762 |
| P-Rab12 S106 | Abcam | Ab256487 | 1:1000 | AB_2884880 |
| Atg16L1 | Cell Signaling Technologies | 8089S | 1:2000 | AB_10950320 |
| LC3B | Thermo Fisher Scientific | PA1-46286 | 1:1000 | AB_2234770 |
| GABARAP | Cell Signaling Technologies | 13733S | 1:1000 | AB_2798306 |
| FIP200 | Cell Signaling Technologies | 12436S | 1:2000 | AB_2797913 |
| LAMP1 (1D4B) | DSHB | AB-528127 | 1:12000  1:400 | AB_2134500 |
| PDI | Cell Signaling Technologies | 2446S | 1:1000 | AB_2298935 |
| GM130 | BD Biosciences | 610822 | 1:1000  1:200 | AB_398141 |
| HA | Sigma-Aldrich/Roche | 12013819001 | 1:1000 | AB_390917 |
| HA | Cell Signaling Technologies | 3724S | 1:100 | AB_1549585 |
| Rabbit IgG (HRP) | Cell Signaling Technologies | 7074S | 1:2000 | AB_2099233 |
| Mouse IgG (HRP) | Cell Signaling Technologies | 7076S | 1:2000 | AB_330924 |
| Rat IgG (HRP) | Cell Signaling Technologies | 7077S | 1:2000 | AB_10694715 |
| Biotin (HRP) | Cell Signaling Technologies | 7075S | 1:4000 | AB_10696897 |
| AlexaFluor 488 anti-rat | Invitrogen | A21208 | 1:600 | AB_2535794 |
| AlexaFluor 568 anti-mouse | Invitrogen | A10037 | 1:600 | AB_11180865 |
| AlexaFluor 647 anti-rabbit | Invitrogen | A31573 | 1:600 | AB_2536183 |
