# Supplementary material for: A STING–CASM–GABARAP pathway activates LRRK2 at lysosomes
Source: J Cell Biol. 2025 Jan 15;224(2):e202310150. doi: 10.1083/jcb.202310150 (PMC11734622; doi:10.1083/jcb.202310150)
Supplement: Table S6 — shows the summary of siRNAs used in this study. [file jcb_202310150_tables6.docx]

**Table S6: Summary of siRNAs used in this study**

| **siRNA** | **Horizon Biosciences Product Number** |
| --- | --- |
| Negative Control | D-001206-13-05 |
| Lrrk2 | M-049666-01-0005 |
| Rab10 | M-040862-01-0005 |
| Rab12 | M-040865-01-0005 |
| Atg3 | M-048439-02-0005 |
| Atg16L1 | M-051699-01-0005 |
| Map1lc3a | M-056203-00-0005 |
| Map1lc3b | M-040989-01-0005 |
| Gabarap | M-041776-01-0005 |
| GabarapL1 | M-040444-01-0005 |
| GabarapL2 | M-059605-01-0005 |
