# Supplementary figures and images for: A STING–CASM–GABARAP pathway activates LRRK2 at lysosomes
Source: J Cell Biol. 2025 Jan 15;224(2):e202310150. doi: 10.1083/jcb.202310150 (PMC11734622; doi:10.1083/jcb.202310150)

**FIGURE 1**

**B**

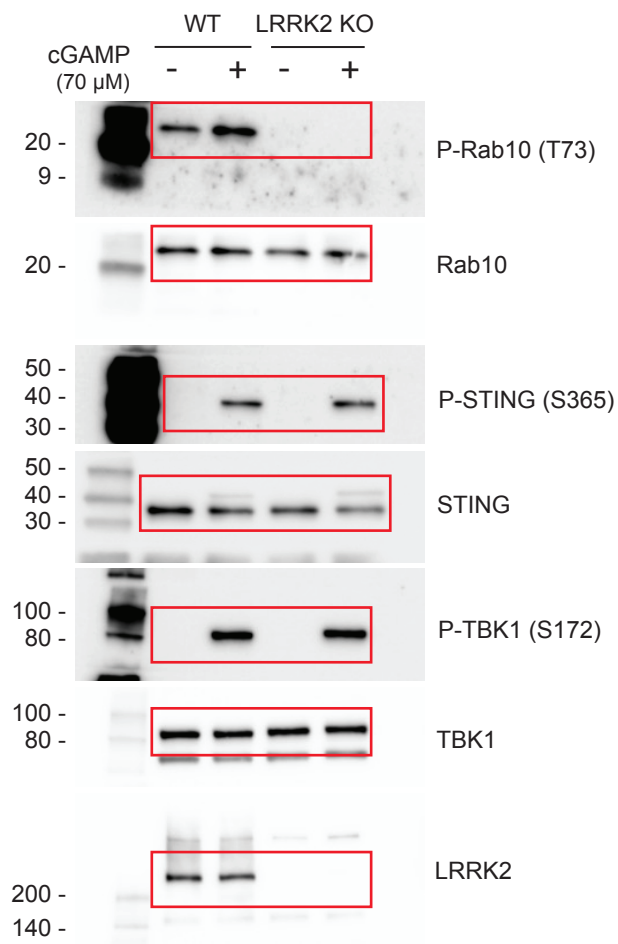

**F**

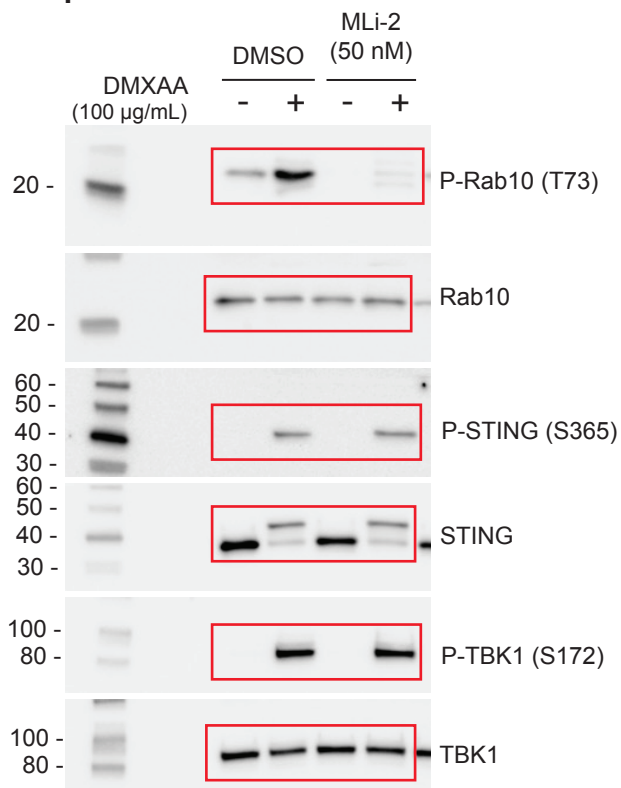

**D**

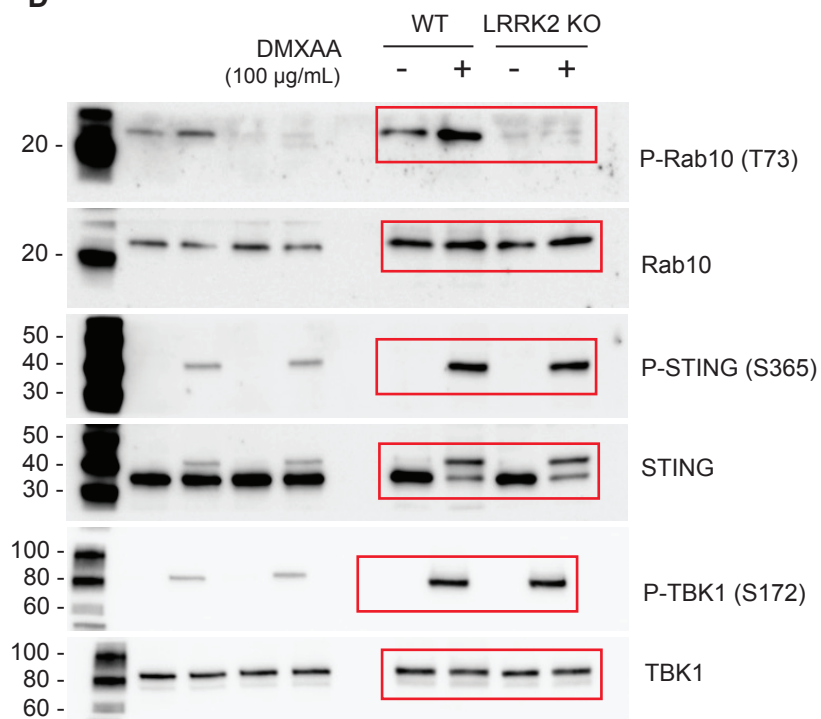

**G**

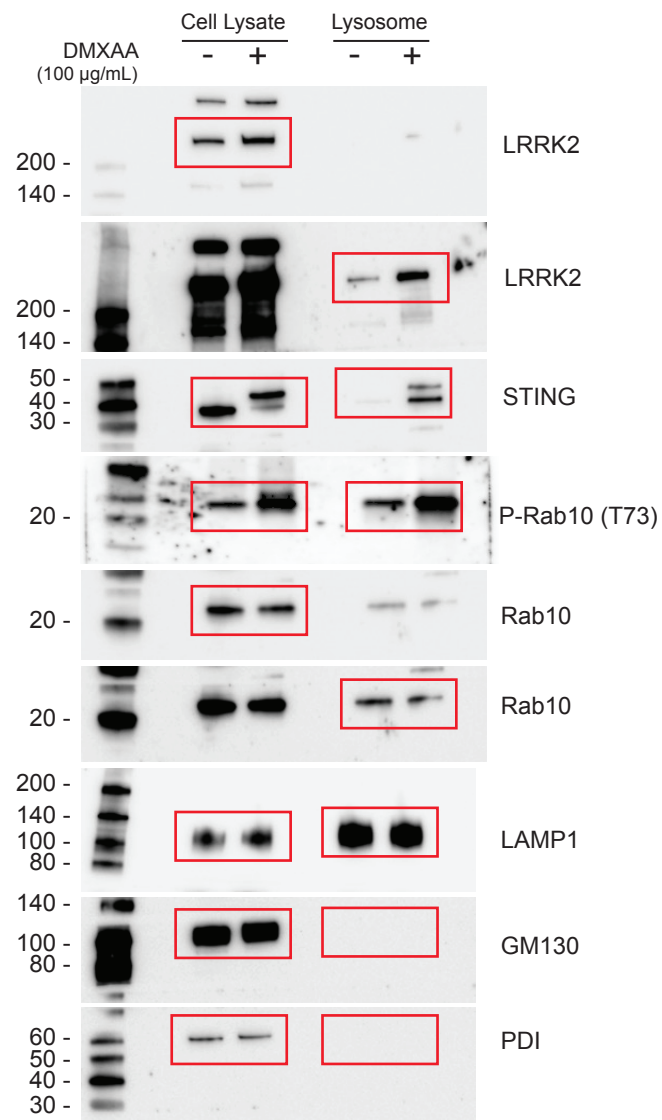

Supplement: SourceData F1 — is the source file for Fig. 1. [file jcb_202310150_sourcedataf1.pdf]

FIGURE 2

A

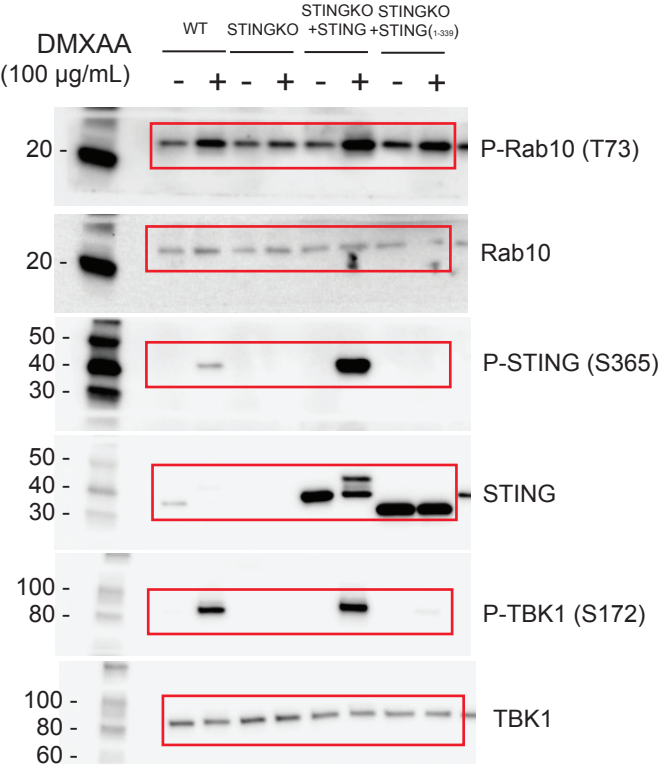

B

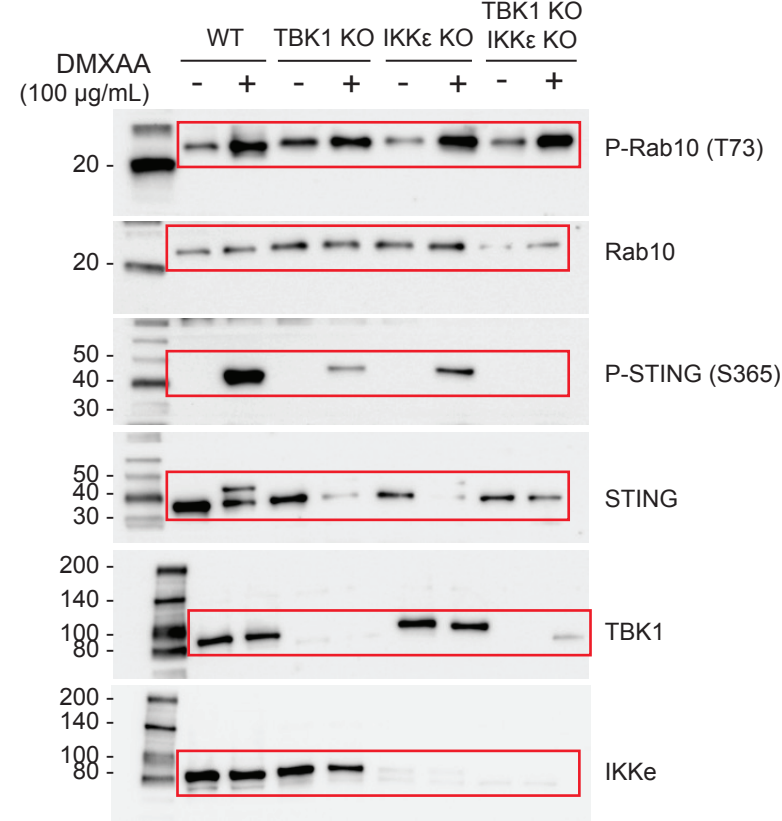

Supplement: SourceData F2 — is the source file for Fig. 2. [file jcb_202310150_sourcedataf2.pdf]

FIGURE 3

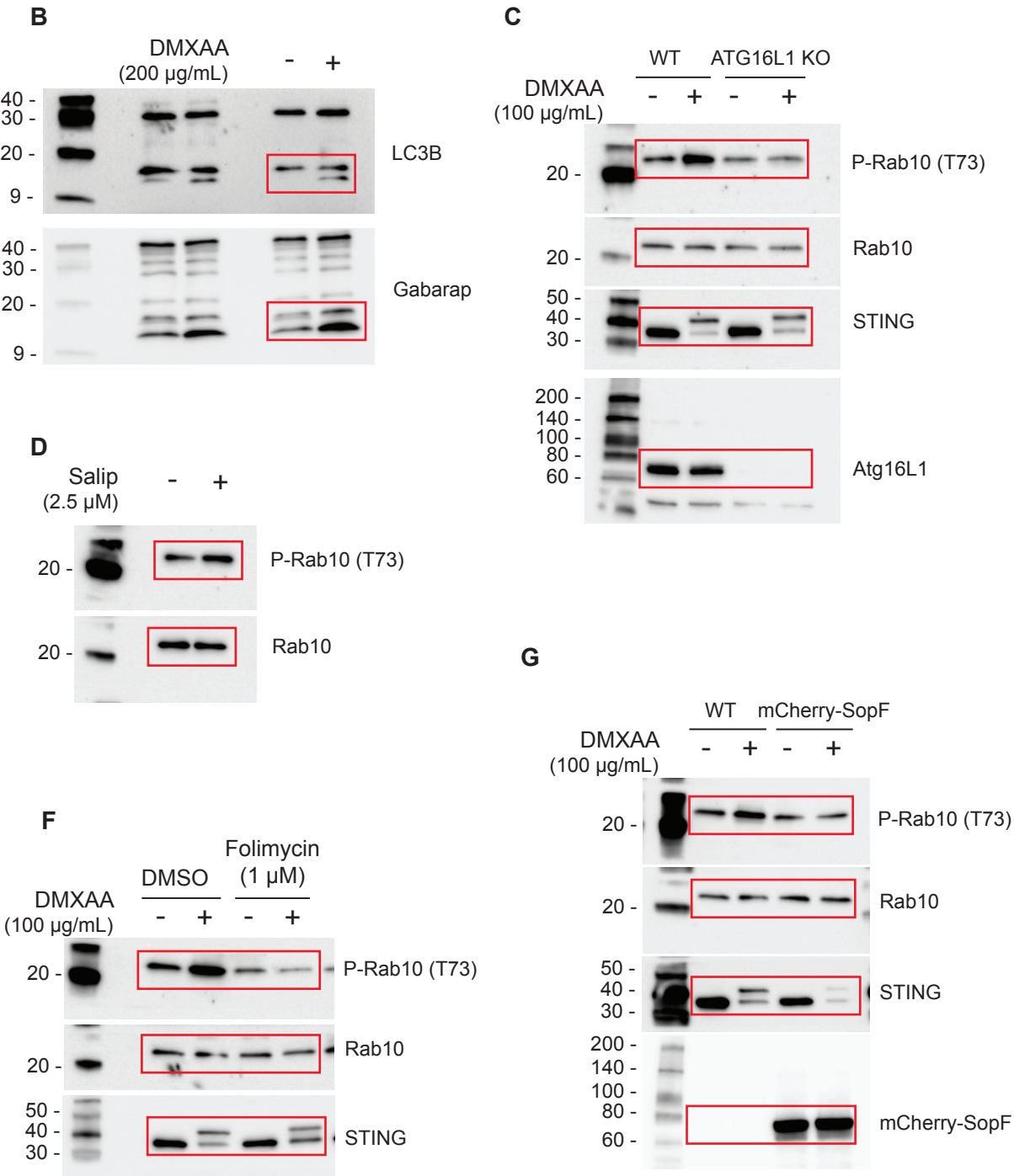

Supplement: SourceData F3 — is the source file for Fig. 3. [file jcb_202310150_sourcedataf3.pdf]

FIGURE 4

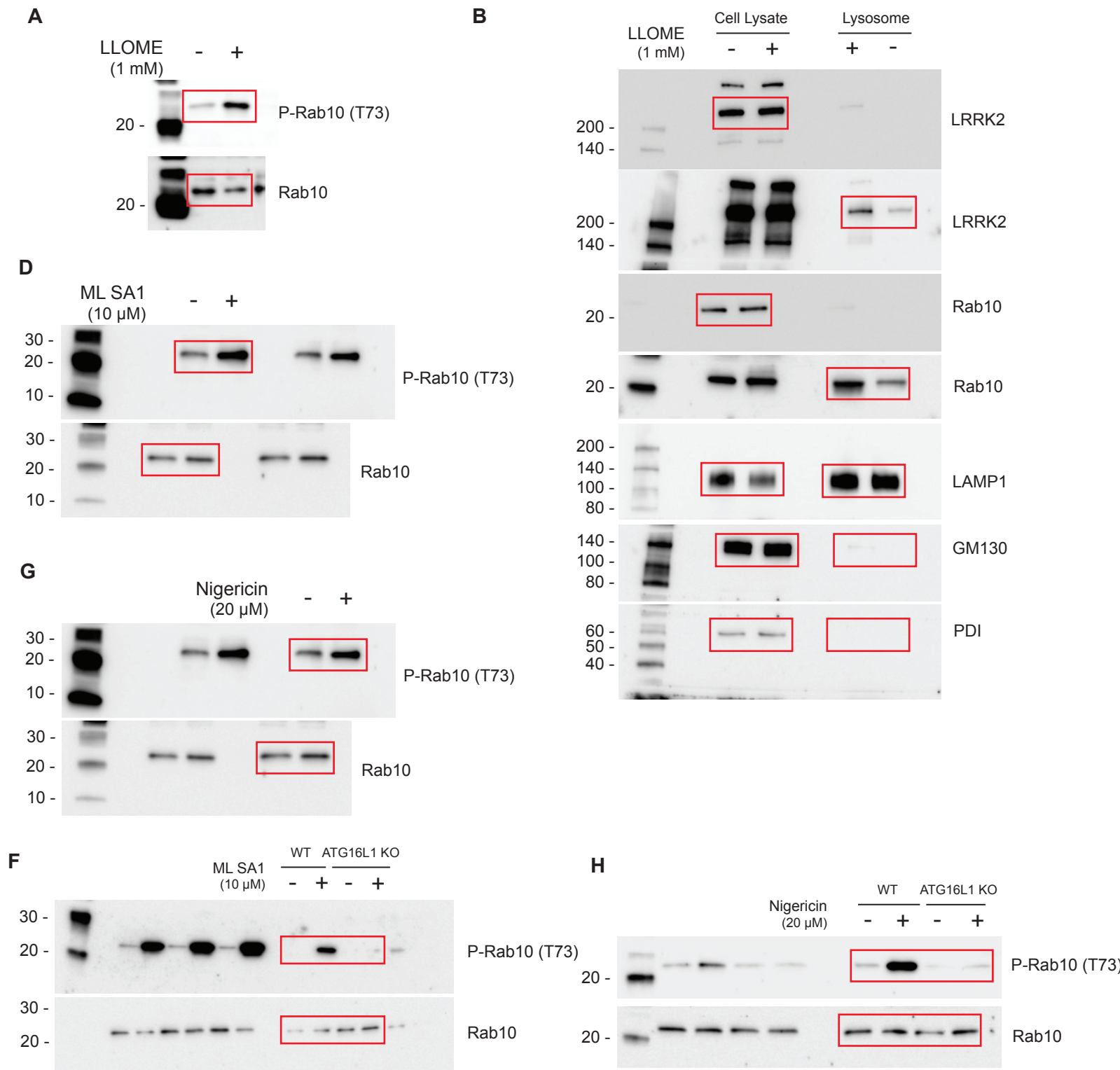

Supplement: SourceData F4 — is the source file for Fig. 4. [file jcb_202310150_sourcedataf4.pdf]

FIGURE 5

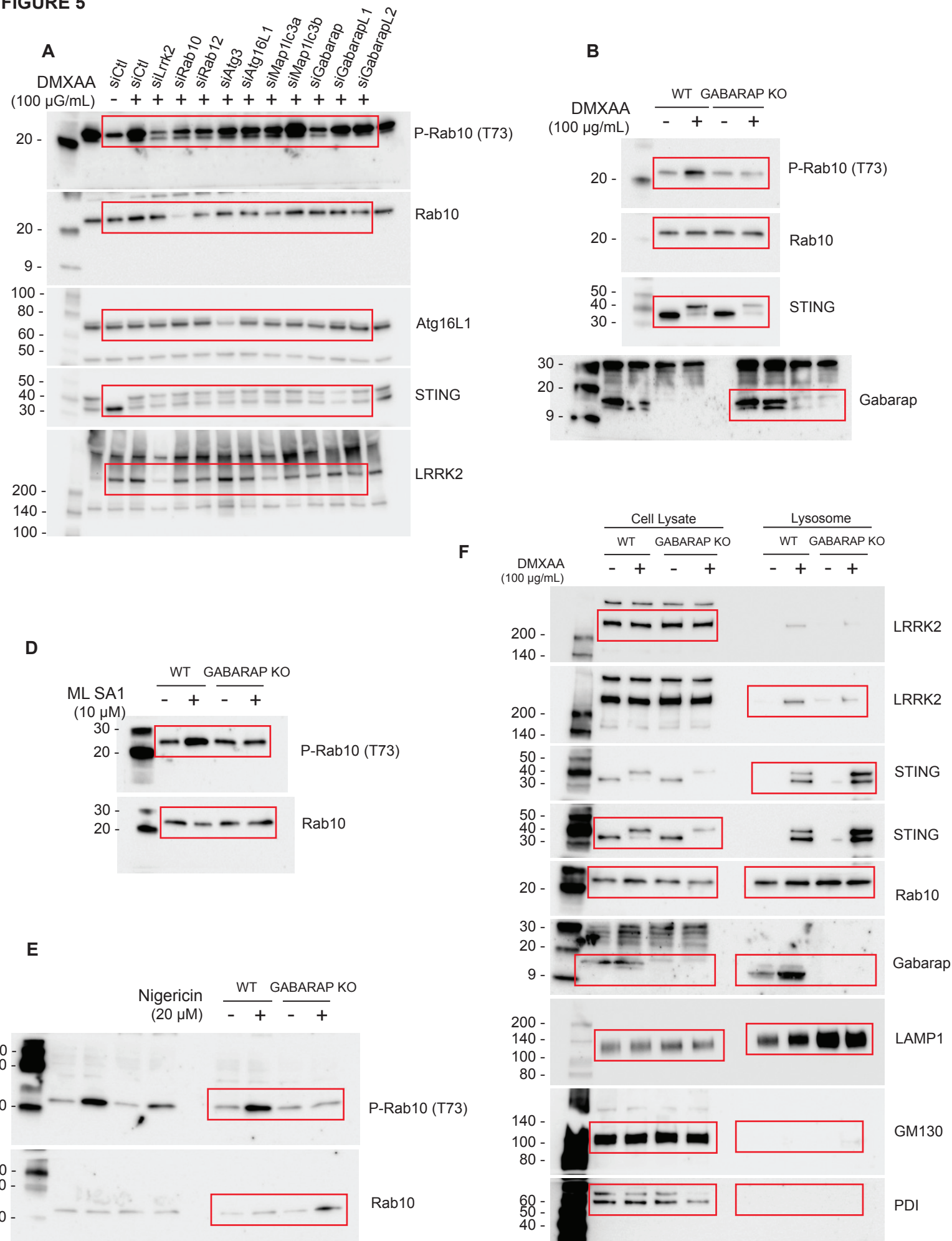

Supplement: SourceData F5 — is the source file for Fig. 5. [file jcb_202310150_sourcedataf5.pdf]

FIGURE 6

A

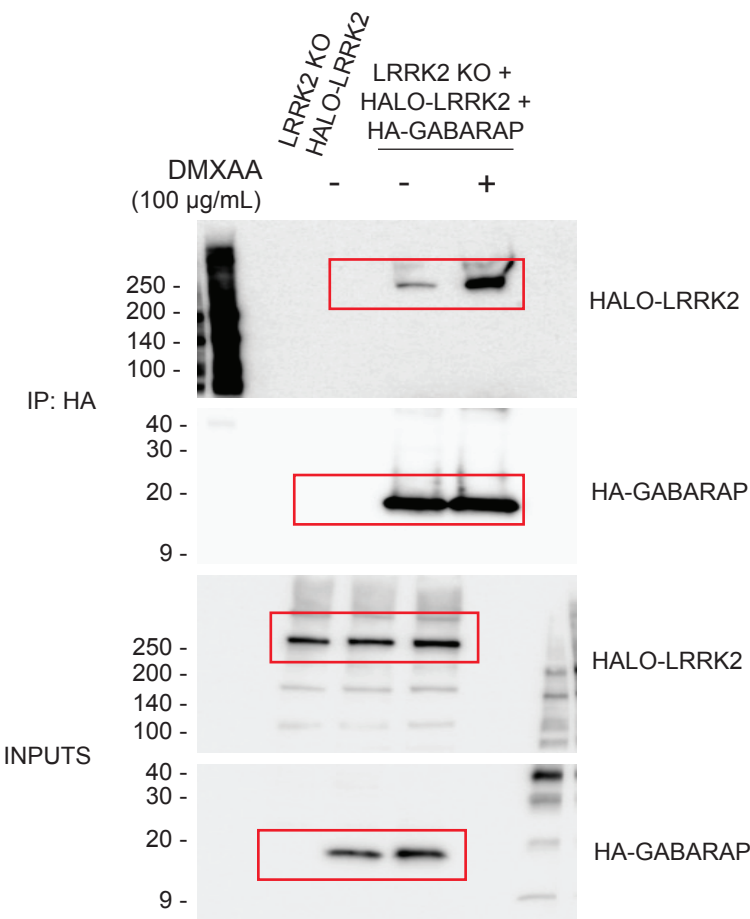

Supplement: SourceData F6 — is the source file for Fig. 6. [file jcb_202310150_sourcedataf6.pdf]

FIGURE 7

A

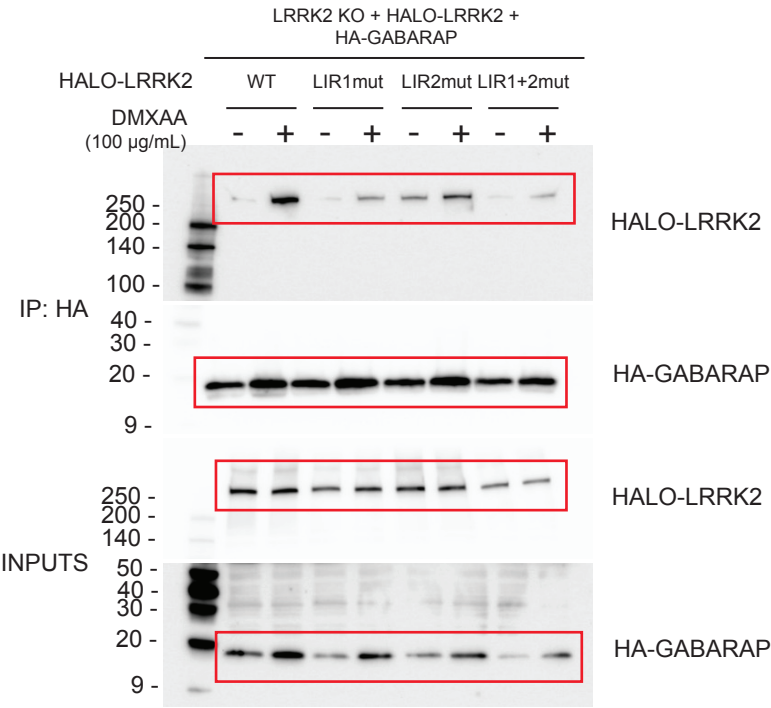

C

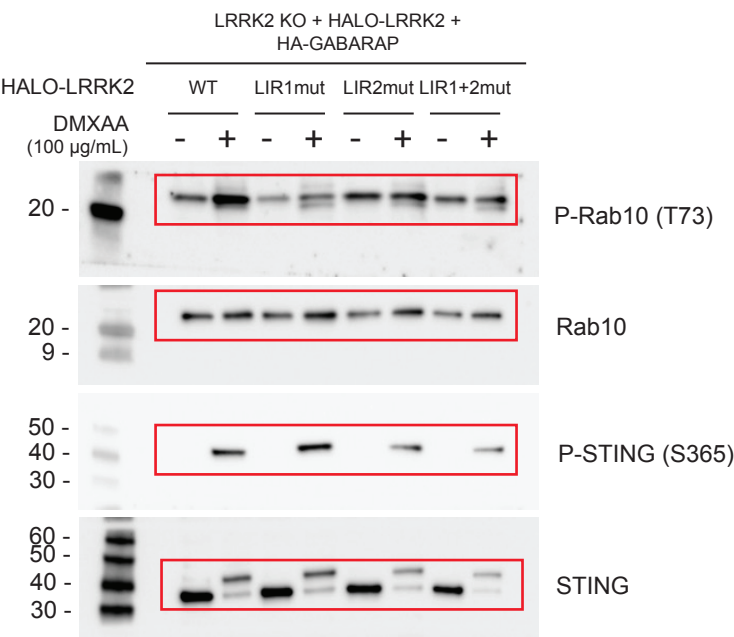

FIGURE 7

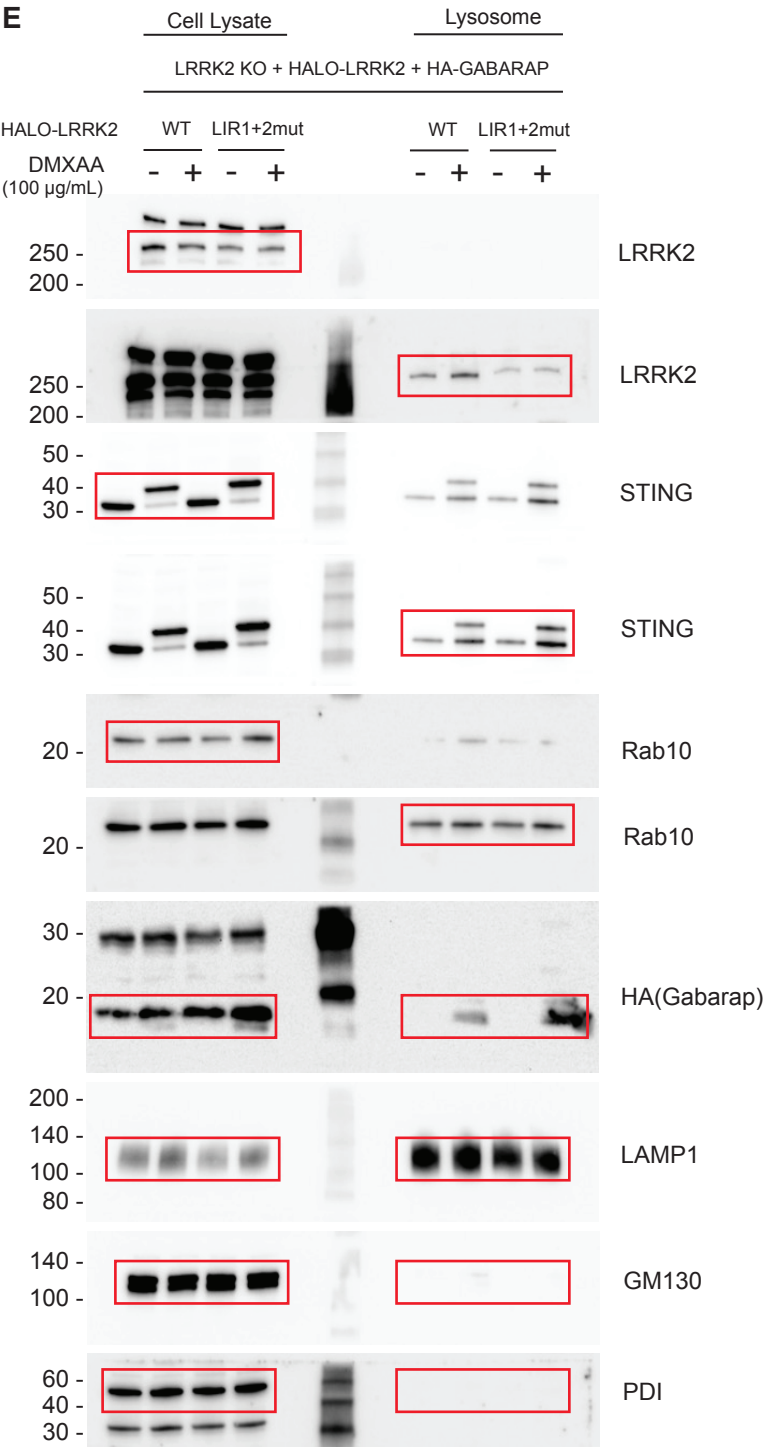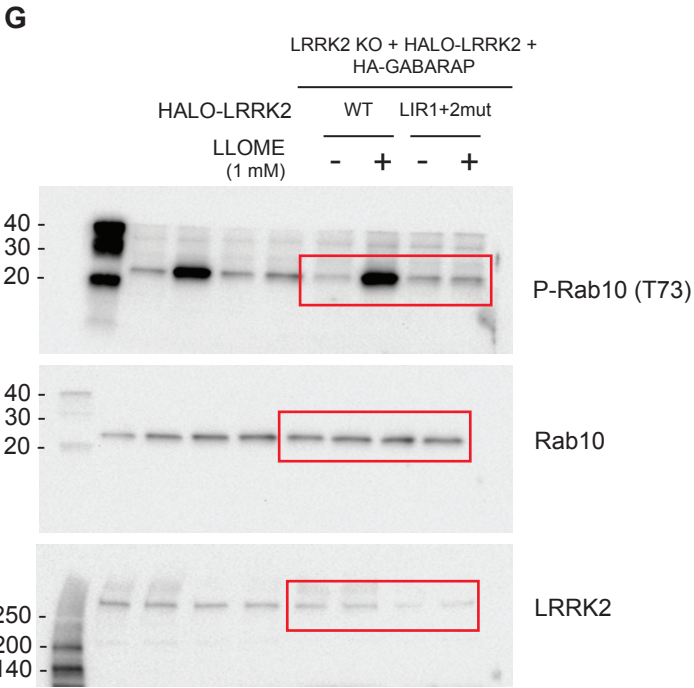

Supplement: SourceData F7 — is the source file for Fig. 7. [file jcb_202310150_sourcedataf7.pdf]

**FIGURE S1**

**A**

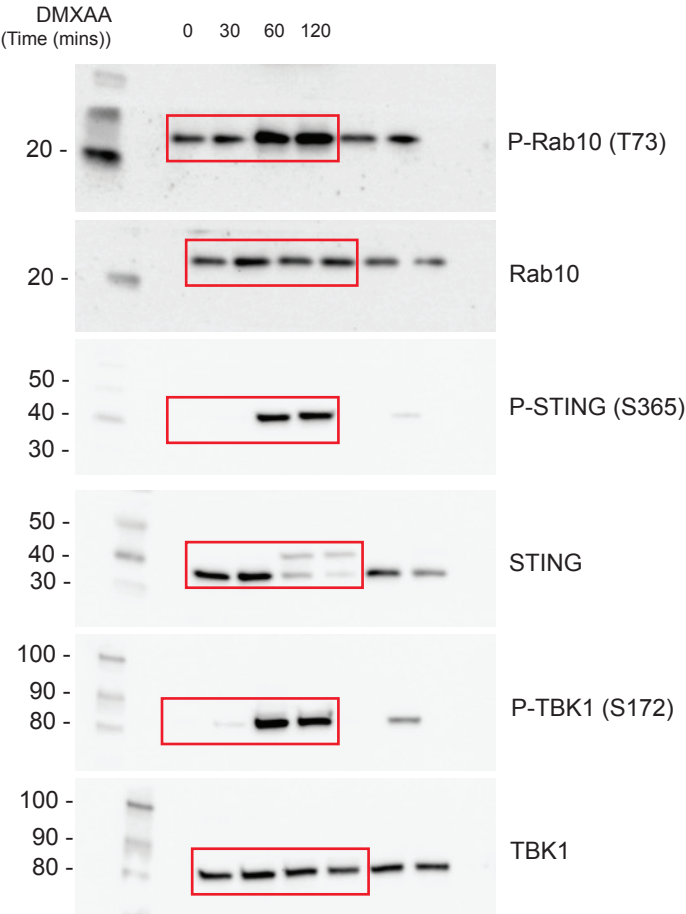

**B**

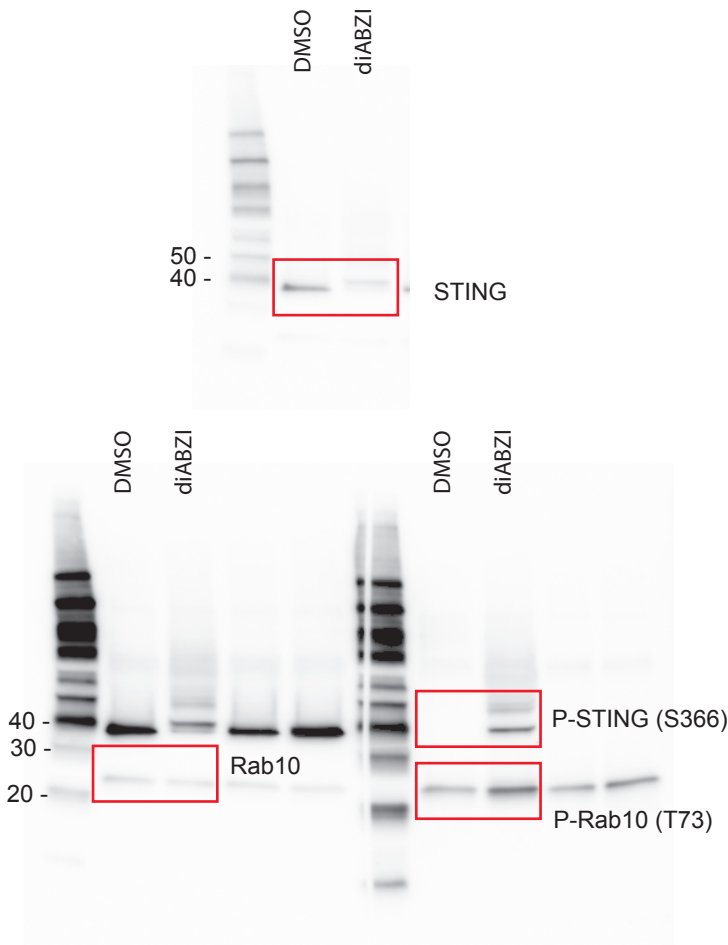

**C**

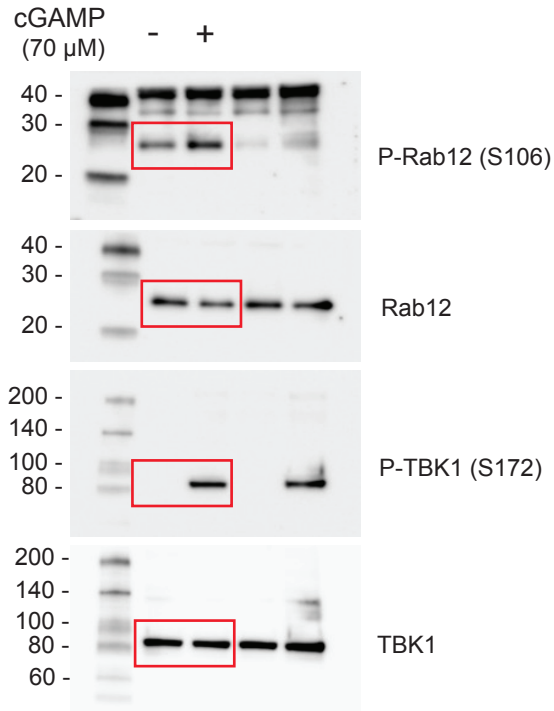

**D**

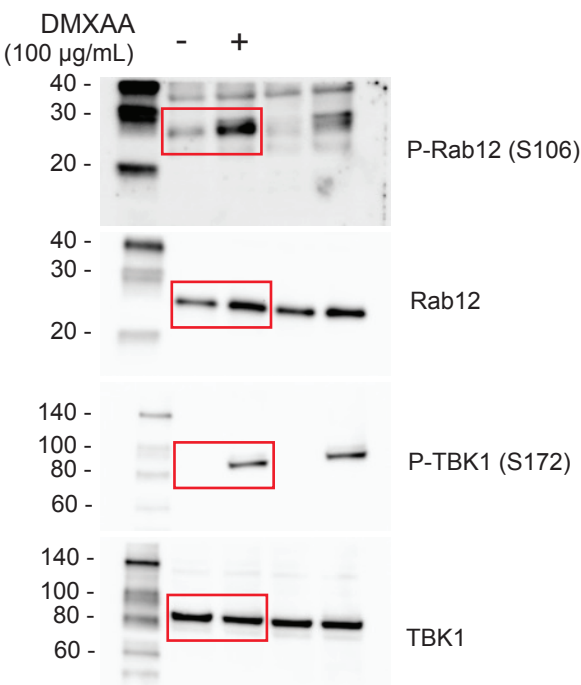

FIGURE S1

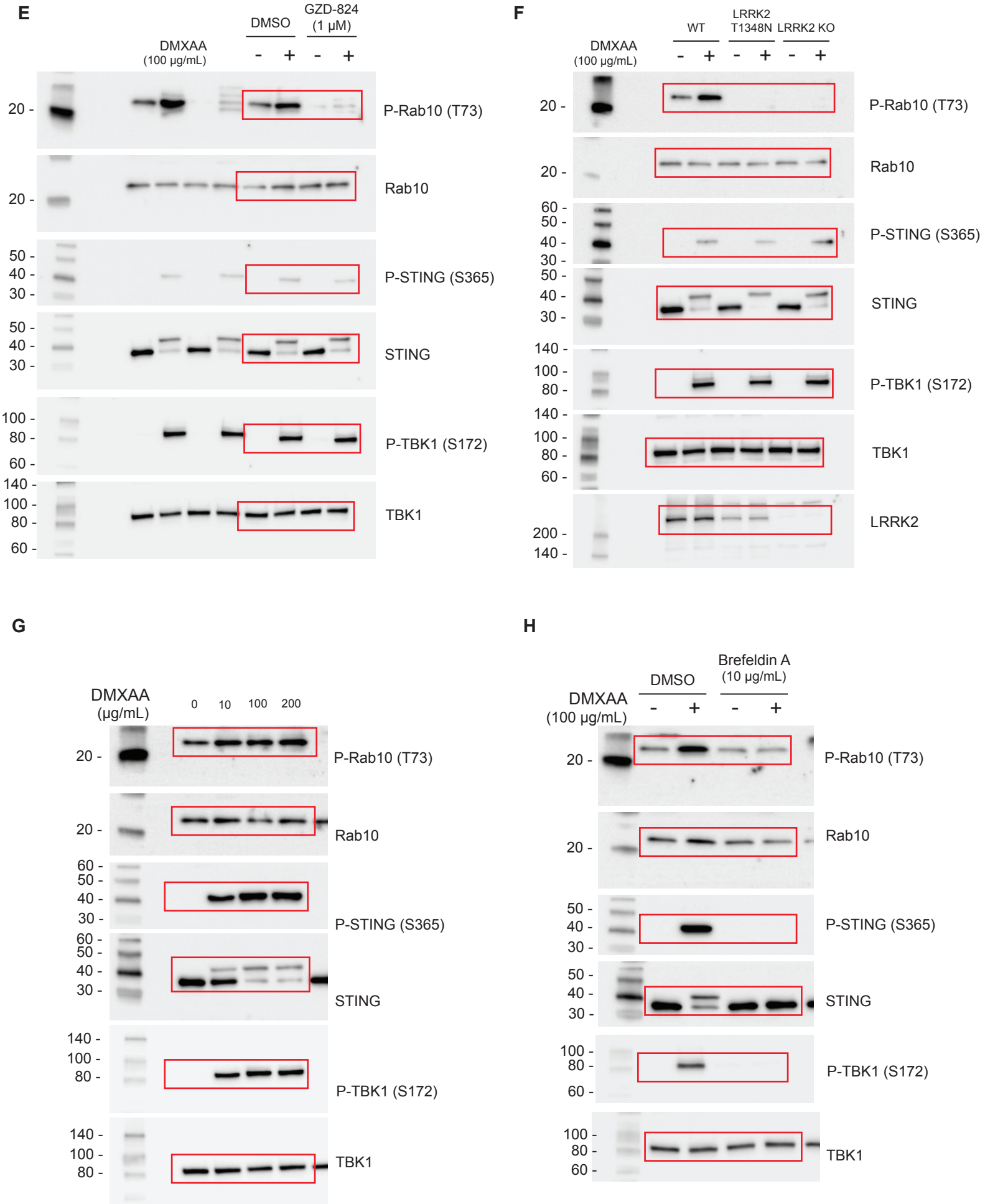

Supplement: SourceData FS1 — is the source file for Fig. S1. [file jcb_202310150_sourcedatafs1.pdf]

FIGURE S2

A

DMSO

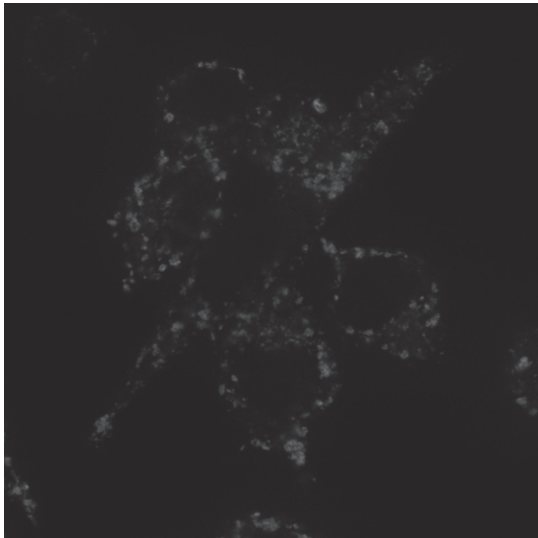

DMXAA  
(100  $\mu$ g/mL)

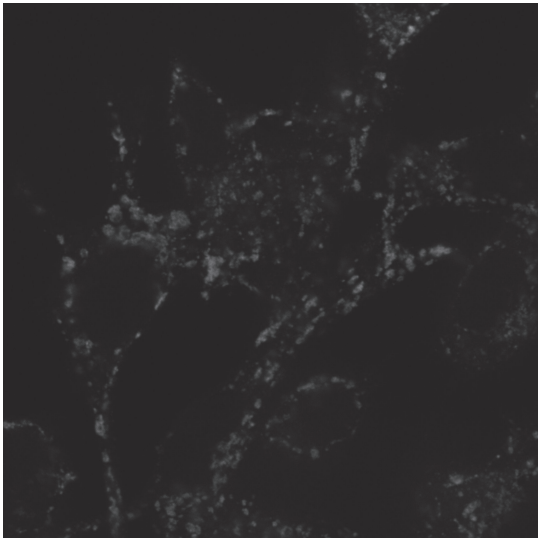

B

DMSO

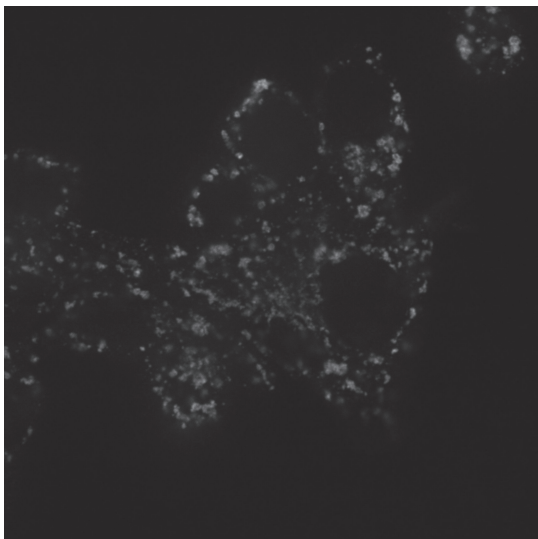

LLOME  
(1 mM)

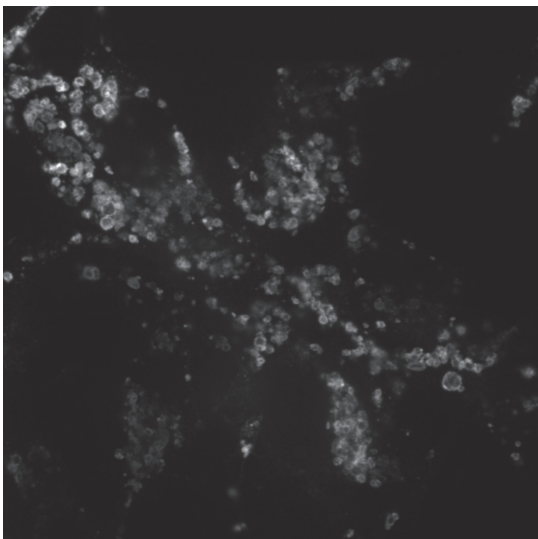

Supplement: SourceData FS2 — is the source file for Fig. S2. [file jcb_202310150_sourcedatafs2.pdf]

**FIGURE S3**

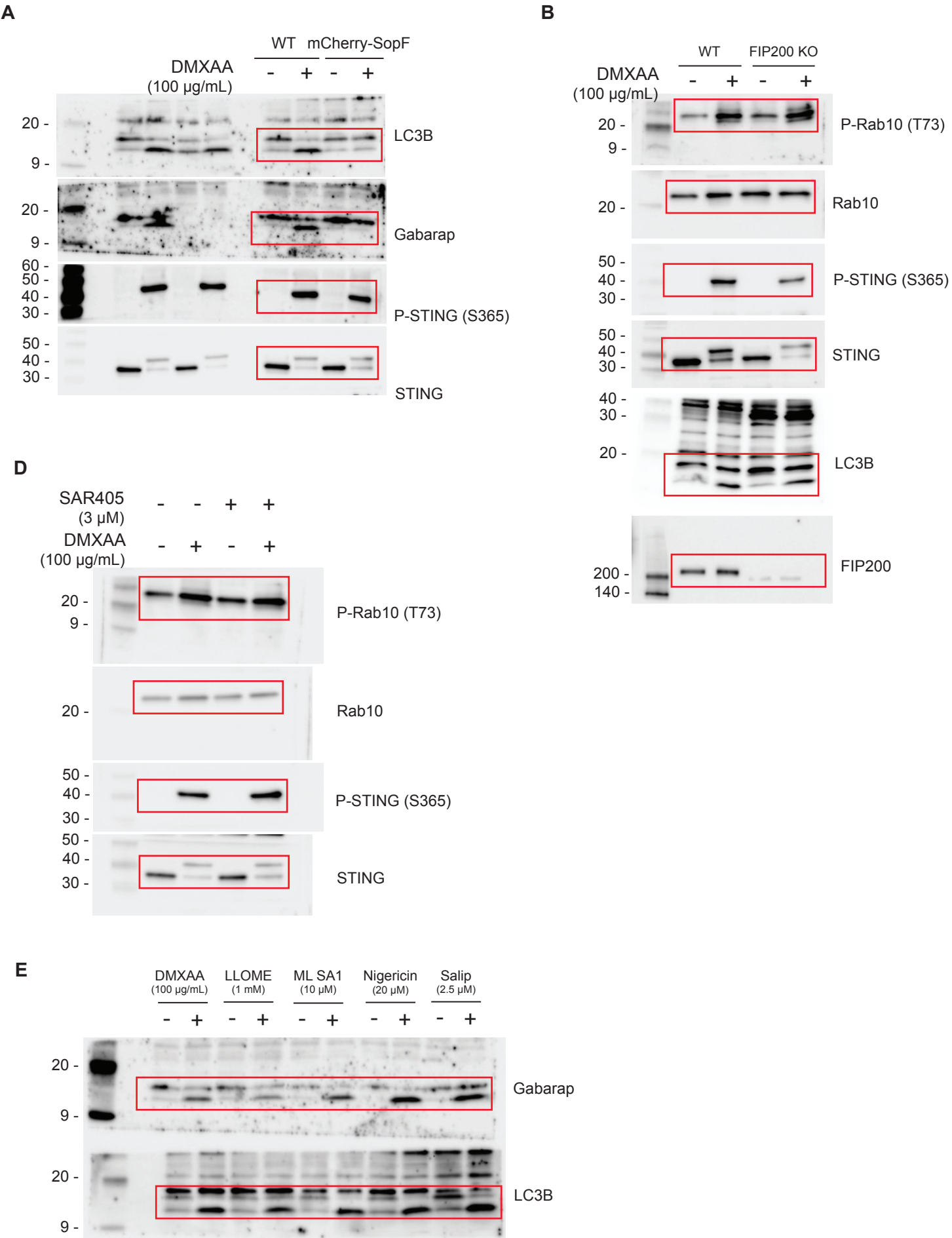

Supplement: SourceData FS3 — is the source file for Fig. S3. [file jcb_202310150_sourcedatafs3.pdf]

FIGURE S4

A

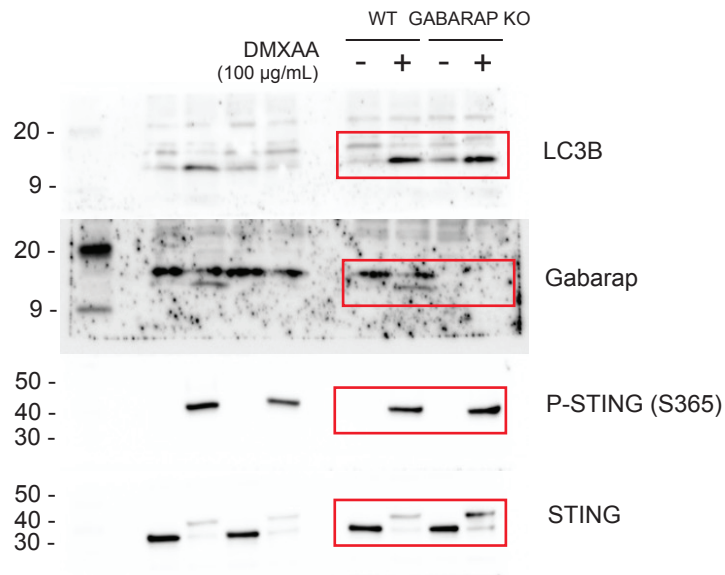

B

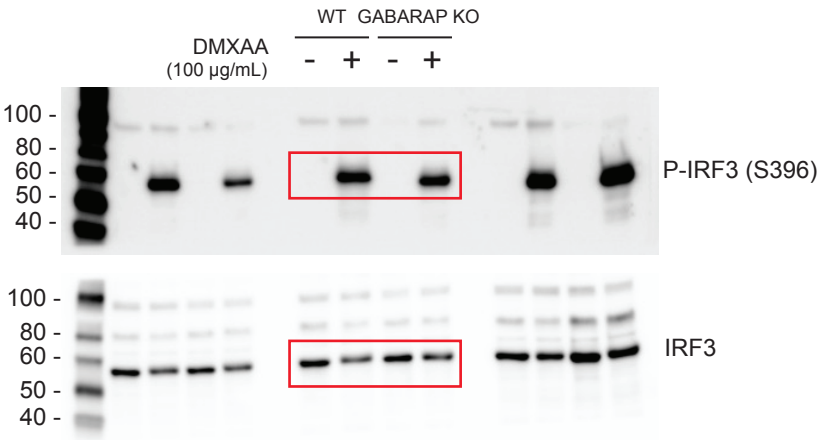

C

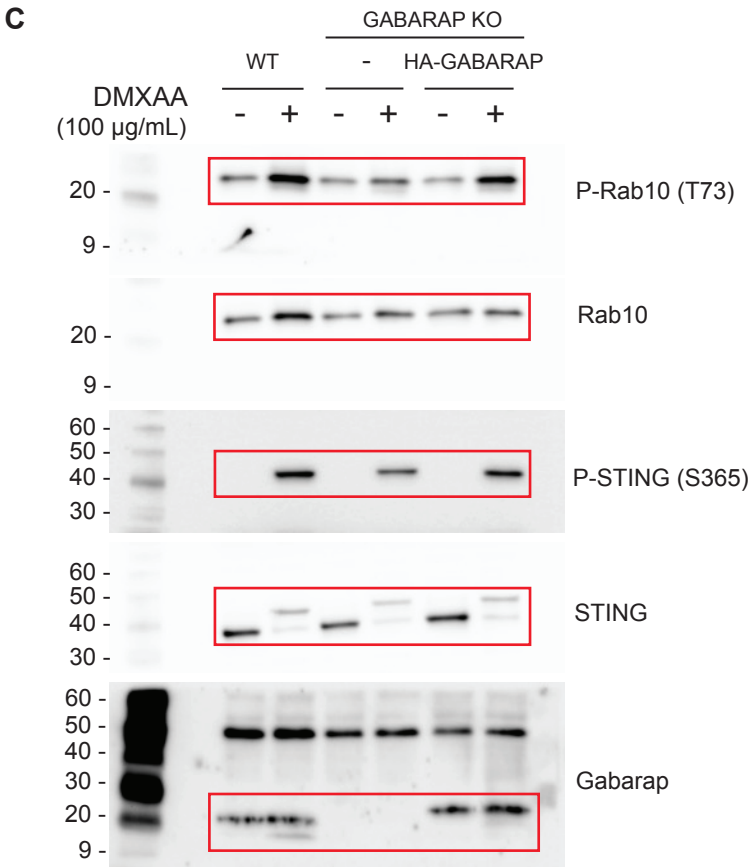

D

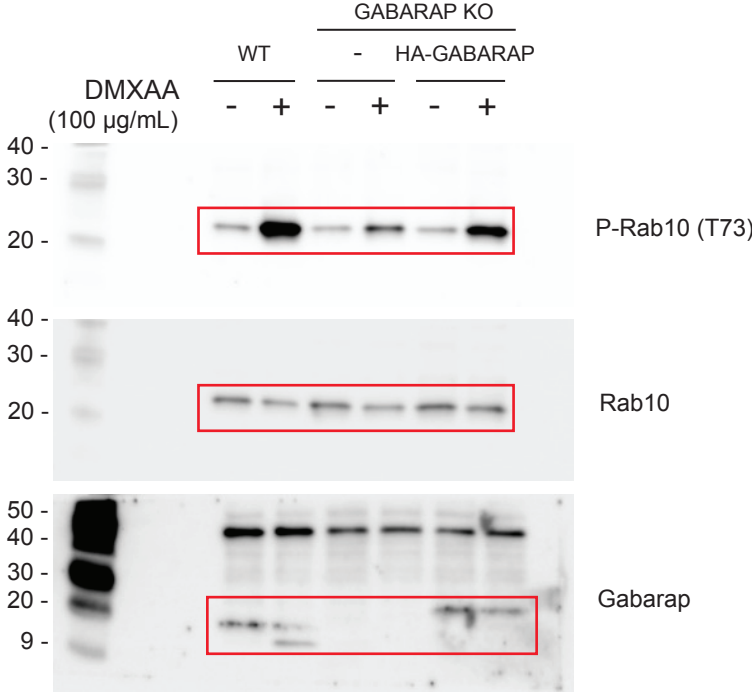

Supplement: SourceData FS4 — is the source file for Fig. S4. [file jcb_202310150_sourcedatafs4.pdf]

FIGURE S5

A

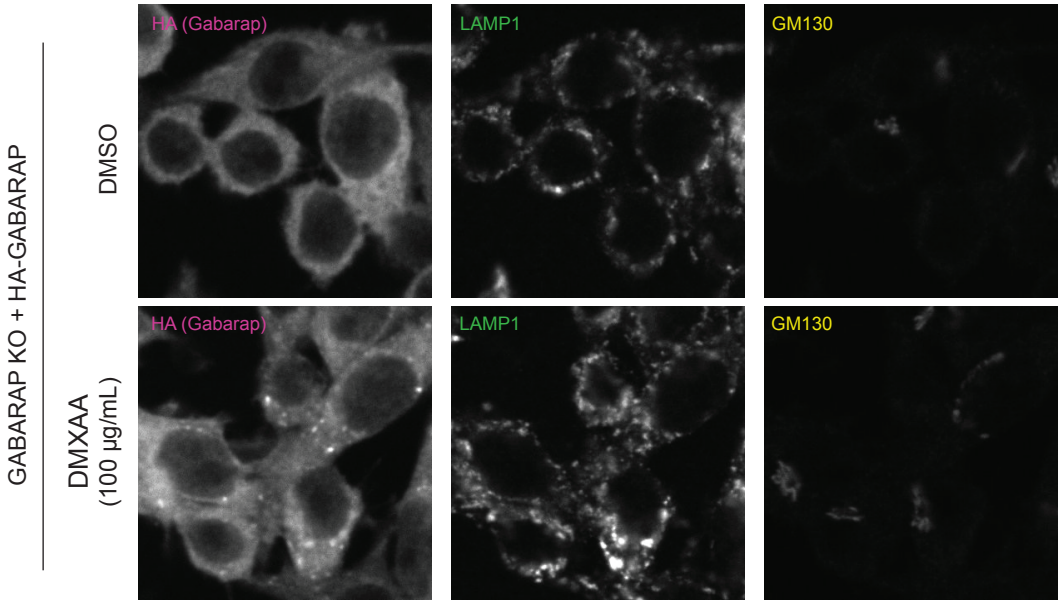

B

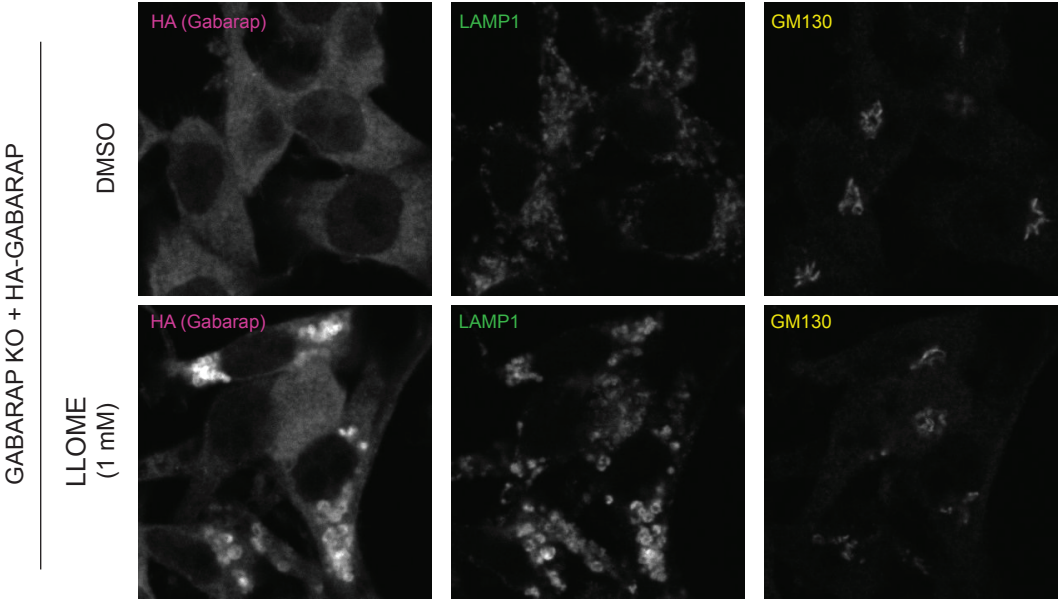

FIGURE S5

C

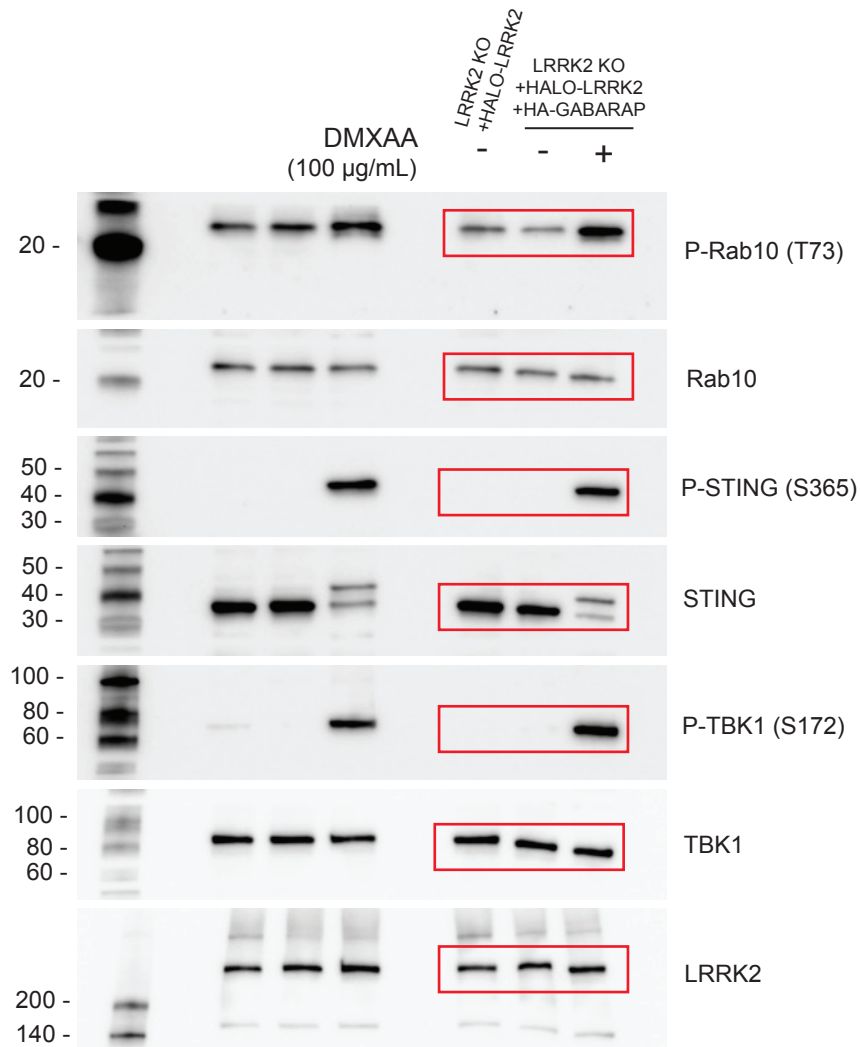

Supplement: SourceData FS5 — is the source file for Fig. S5. [file jcb_202310150_sourcedatafs5.pdf]
